# Integrated Gene Expression Data-Driven Identification of Molecular Signatures, Prognostic Biomarkers, and Drug Targets for Glioblastoma

**DOI:** 10.1155/2024/6810200

**Published:** 2024-08-16

**Authors:** Md. Wasim Alom, Md. Delowar Kobir Jibon, Md. Omar Faruqe, Md. Siddikur Rahman, Farzana Akter, Aslam Ali, Md Motiur Rahman

**Affiliations:** ^1^ Department of Genetic Engineering and Biotechnology University of Rajshahi, Rajshahi 6205, Bangladesh; ^2^ Department of Computer Science and Engineering University of Rajshahi, Rajshahi 6205, Bangladesh

**Keywords:** biomarker, differentially expressed genes, dynamics, glioblastoma, molecular docking

## Abstract

Glioblastoma (GBM) is a highly prevalent and deadly brain tumor with high mortality rates, especially among adults. Despite extensive research, the underlying mechanisms driving its progression remain poorly understood. Computational analysis offers a powerful approach to explore potential prognostic biomarkers, drug targets, and therapeutic agents for GBM. In this study, we utilized three gene expression datasets from the Gene Expression Omnibus (GEO) database to identify differentially expressed genes (DEGs) associated with GBM progression. Our goal was to uncover key molecular players implicated in GBM pathogenesis and potential avenues for targeted therapy. Analysis of the gene expression datasets revealed a total of 78 common DEGs that are potentially involved in GBM progression. Through further investigation, we identified nine hub DEGs that are highly interconnected in protein–protein interaction (PPI) networks, indicating their central role in GBM biology. Gene Ontology (GO) and pathway enrichment analyses provided insights into the biological processes and immunological pathways influenced by these DEGs. Among the nine identified DEGs, survival analysis demonstrated that increased expression of GMFG correlated with decreased patient survival rates in GBM, suggesting its potential as a prognostic biomarker and preventive target for GBM. Furthermore, molecular docking and ADMET analysis identified two compounds from the NIH clinical collection that showed promising interactions with the GMFG protein. Besides, a 100 nanosecond molecular dynamics (MD) simulation evaluated the conformational changes and the binding strength. Our study highlights the potential of GMFG as both a prognostic biomarker and a therapeutic target for GBM. The identification of GMFG and its associated pathways provides valuable insights into the molecular mechanisms driving GBM progression. Moreover, the identification of candidate compounds with potential interactions with GMFG offers exciting possibilities for targeted therapy development. However, further laboratory experiments are required to validate the role of GMFG in GBM pathogenesis and to assess the efficacy of potential therapeutic agents targeting this molecule.

## 1. Introduction

Glioblastoma (GBM) is a highly aggressive and deadly form of brain cancer that arises from astrocytes, the supportive cells of the brain. It is also referred to as GBM multiforme (GBM) [1]. It is the most common primary brain tumor in adults and accounts for approximately 47% of all malignant brain tumors [2]. The prognosis for GBM patients is extremely poor, with a survival of 15 to 23 months and a less than 6% chance of surviving beyond 5 years after diagnosis [3]. The incidence of GBM is particularly prominent in North America, Australia, and northern and western Europe [4]. It has a yearly incidence rate of 4.23 per 100,000 individuals [5]. According to experimental data, GBM can develop into various types of tumors and contain a subset of highly tumourigenic cells known as GBM stem cells, which are believed to be responsible for recurrent GBM [6, 7]. GBMs can be classified as primary or secondary GBMs, as mentioned earlier [8, 9]. Primary GBM, which develops rapidly in older people (mean age 55) without prior brain malignancies, accounts for nearly 90% of cases and is characterized by mutations in IDH1, PDGFRA abnormalities, EGFR overexpression, PTEN mutation, and chromosome loss [10–12]. In contrast, secondary GBM, which accounts for approximately 10% of cases, evolves from preexisting low-grade gliomas and is marked by mutations in IDH1, IDH2, MGMT, TP53, and alterations in 19q. Molecular markers, such as EGFR, PDGFRA, NF1, IDH1, MGMT, p53, and PTEN are utilized for characterizing GBM [13–15].

Among the molecular biomarkers identified in GBM, the IDH1, MGMT, and EGFR were demonstrated to have clinical significance [16–18]. IDH1 mutations are typically associated with younger patients and secondary GBM, often accompanied by TP53 mutations, and are incorporated into WHO diagnostic guidelines as a positive prognostic indicator [16, 19, 20]. MGMT promoter methylation, while not included in current WHO classifications, is a crucial prognostic marker that predicts response to alkylating agents like temozolomide [17, 21]. Promoter methylation-induced MGMT silencing enhances temozolomide's cytotoxic effects, potentially increasing patient survival [22]. EGFR abnormalities, driven by amplification or the EGFRvIII mutation, are linked to higher tumor malignancy and poorer prognosis [18]. EGFR serves both as a prognostic marker and a therapeutic target, with anti-EGFR therapies like Gefitinib and Erlotinib being tested in clinical trials to inhibit its downstream signaling by preventing tyrosine residue phosphorylation [23, 24]. While IDH1 mutations offer a better prognosis, they are less prevalent in primary GBM, limiting their applicability in a significant portion of patients [25]. Additionally, although MGMT promoter methylation can forecast response to temozolomide, not all patients with methylated MGMT experience equal benefits, suggesting the influence of other factors on treatment response [26]. Moreover, clinical trials of EGFR-targeted therapies have demonstrated limited efficacy, partly due to tumor heterogeneity and acquired resistance mechanisms [27]. The intricate interplay between these biomarkers and other molecular alterations in GBM necessitates further investigation to fully understand their clinical relevance and therapeutic implications. Exploring more biomarkers beyond IDH1, MGMT, and EGFR could reveal new GBM subtypes, improving personalized treatment and patient outcomes. Integrating multiple biomarkers may enhance precision medicine, tailoring therapies to each tumor's unique profile [28]. Biomarker discovery not only identifies new treatment targets but also guides the development of innovative therapies, making ongoing efforts essential for advancing GBM understanding and patient care.

For most GBM patients, no specific risk factors have been identified. The only known external environmental risk factor for glioma is exposure to ionizing radiation [29, 30]. Other factors, such as viral triggers (human cytomegalovirus) [31], adolescent obesity [32], and a family history of cancer [33], are still under investigation. Current research is focused on identifying germline polymorphisms associated with an increased risk of GBM, as genetic factors play a role in determining the level of risk associated with these exposures [30]. The currently available treatments for GBM include maximal resection (complete resection is very rare, because these tumors spread throughout the body) followed by radiotherapy with concurrent adjuvant therapies, such as temozolomide (TMZ). Bevacizumab, which inhibits circulating vascular endothelial growth factor (VEGF), is often recommended for patients with progressive cancer and has recently been used in combination with lomustine (CCNU) [34]. Despite numerous efforts, there has been no improvement in the survival rates of most GBM patients, and GBM patients suffer recurrence as a result of molecular heterogeneity and the challenge of drug penetration across the blood–brain barrier (BBB). However, recent developments in genomics and transcriptomics have led to the discovery of specific molecular signatures of GBM that enable us to better understand the molecular mechanism of GBM. Identifying the crucial molecular signatures, biomarkers, and therapeutic targets in GBM could significantly improve treatment strategies and decrease fatalities. A prognostic biomarker refers to a genetic marker that forecasts the probability of a forthcoming clinical event, recurrence of a disease, or its progression within a population. The biological features of biomarkers help predict the course of a disease or the response to treatment among patients.

The Gene Expression Omnibus (GEO) database serves as a global online repository for microarray gene expression data and contains a wide range of readily accessible functional genomics datasets. The exploration of microarray data can provide insights into the pathology and molecular mechanisms underlying GBM. Therefore, we analyzed microarray data using *in silico* techniques to examine the role of genes in pathogenic processes. The major objective of this study was to identify potential biomarkers, molecular signatures, and therapeutic agents that could contribute to the early detection of GBM and serve as molecular targets for drug candidates.

## 2. Materials and Methods

Publicly accessible data were analyzed by the following methods to meet the aim set for this study.

### 2.1. Data Collection and Retrieval

To identify the significant components of the molecular pathways associated with GBM, we retrieved three gene expression datasets (Table 1) from the GEO database [35]. The datasets were chosen, as they include samples from both GBM tissues and normal brain tissues, allowing for direct comparisons with high-quality data. The datasets focus specifically on GBM, excluding other types of brain tumors or unrelated diseases, ensuring relevance to the study. Each dataset contains a sufficient number of samples to ensure robust statistical power. The use of consistent platforms, namely, GPL570 and GPL8300, helps minimize technical variability and enhances the reliability of the findings. Details of the selected datasets and their DEGs are shown in Table 1.

### 2.2. Identification of Differentially Expressed Genes (DEGs)

The GEO 2R (https://www.ncbi.nlm.nih.gov/geo/geo2r/), an interactive web server for analyzing microarray datasets, was used to identify DEGs in the GSE50161, GSE12657, and GSE15824 datasets by comparing normal and disease samples. The datasets were analyzed and normalized by the Limma [36] package to identify DEGs in GBM. For all the datasets, a threshold of *p* value < 0.05 and |logFC| > 1.0 was set to determine significant DEGs. Common genes were also identified from the datasets via Venn analysis using the web tool jvenn [37].

### 2.3. Hierarchical Clustering

We utilized the Cluster 3.0 tool to conduct clustering analysis on the selected gene expression data and employed another tool called Java TreeView to visualize the resulting analysis. The clustering process started by creating a tab-delimited text file to use as input for the Cluster 3.0 tool. The initial step in hierarchical clustering involves calculating the distance matrix for the gene expression data. After this matrix of distances is computed, the clustering process can begin. The generated CDT file from Cluster 3.0 was the input file for Java TreeView, which produced a dendrogram displaying the hierarchical clustering of genes, including both gene tree and array tree. Hierarchical clustering methods arrange genes into a tree-like formation according to their similarity [38, 39]. Cluster 3.0 represents an enhanced iteration of the Cluster program, utilizing the C Clustering Library [40].

### 2.4. Construction of the PPI Network

Utilizing the STRING (https://string-db.org/) database [41], a protein–protein interaction (PPI) network of proteins encoded by common DEGs was constructed to illustrate how the defined DEGs and proteins physically and functionally interact with each other. We also identified hub genes from the network by using the MCODE algorithm [42]. The generated network file was customized in Cytoscape (https://cytoscape.org) [43].

### 2.5. Gene Ontology (GO) and Pathway Enrichment Analysis

Gene set enrichment analysis (GSEA) is a computational and statistical methodology that determines whether a collection of determined genes exhibits statistical significance under various biological conditions. Pathway annotations were acquired from the Reactome database. GO terms and pathways for the present study were obtained using the EnrichR (https://amp.pharm.mssm.edu/Enrichr/) platform [44]. For all analyses, a *p* value < .05 was considered to indicate statistical significance.

### 2.6. Identification of Transcription Factor (TF) Interactions With Hub DEGs

TFs interact with genes to control gene expression by activating or inactivating transcription. We identified TF-gene interactions with the hub DEGs using the JASPAR [45] and TRANSFAC [46] databases through EnrichR.

### 2.7. Survival Analysis

To examine the prognostic performance of the hub genes in detecting GBM, a multivariate survival analysis of GBM patients was performed based on the expression of the hub genes by using the GEPIA2 web tool (http://gepia2.cancer-pku.cn/) [47]. The significance level was set to a *p* value < 0.05.

### 2.8. Validation of Genes

The selected key DEG was the *GMFG* gene, which was validated through differential expression analysis via GEPIA2. The mRNA expression pattern of the *GMFG* gene in GBM tissue was determined using two servers, UALCAN (http://ualcan.path.uab.edu) and the OncoDB server (http://oncodb.org/), to increase the fidelity of the findings. The GEPIA 2 is an online platform that enables the analysis of RNA sequencing data from The Cancer Genome Atlas (TCGA) and Genotype-Tissue Expression (GTEx) datasets on malignant and normal tissues [47]. The UALCAN web portal enables the cancer research community to analyze and receive cancer transcriptome, proteome, and patient survival data [48]. OncoDB also facilitates the investigation of differential gene expression in malignant tissues and the correlation of gene expression with the clinical outcome of cancer patients [49]. The differential expression of *GMFG* in cancerous conditions was also investigated by analyzing the immunohistochemistry of GBM and healthy cells curated from the Human Protein Atlas database [50].

### 2.9. Structural Preparation of Proteins and Ligands

The crystal structure of human GMFG, PDB ID 3L50, was extracted from the Protein Data Bank database (https://www.rcsb.org) [51]. The structure was selected because it exhibited a lower resolution (<1.90 Å) in its X-ray crystallographic structure and demonstrated better percentile scores in global validation metrics, signifying superior structural quality. Water molecules, native ligands, and heteroatoms present in the crystallized protein structure were removed using BIOVIA Discovery Studio 2019 software [52]. Additional critical factors, such as side-chain geometry, hydrogen correction, and correction of improper bond orders, were addressed and minimized using the GROMOS 43B1 force field of Swiss-PDB Viewer version 4.10 [53].

A total of 692 compounds were included as ligands from the NIH clinical collection; these compounds consisted of molecules with a documented history of use in human clinical trials. The 3D structures of these compounds were retrieved from the PubChem database (https://pubchem.ncbi.nlm.nih.gov/) [54]. The ligands underwent energy minimization using the mmff94 force field within the Open Babel plug-in of the PyRx tool [55].

### 2.10. Molecular Docking

Molecular docking was performed by employing the AutoDock plug-in within PyRx software by loading and converting each ligand and the GMFG receptor from the PDB format to the PDBQT format. Hydrogens with polar characteristics were introduced into the enzyme, and hydrogens with nonpolar characteristics were combined. The grid box for the GMFG enzyme was established as follows: the center points of the box were *X* = 4.8407, *Y* = 27.8057, and *Z* = 12.8898, and the dimensions (Å) were *X* = 46.6266, *Y* = 42.0797, and *Z* = 38.1385. Molecular docking was carried out using AutoDock Vina in PyRx [56]. The binding affinities of the ligands to the receptors were calculated in kcal/mol, with negative values indicating stronger binding. Molecular visualization and nonbonding interactions of the protein–ligand complexes were analyzed utilizing Discovery Studio 2019.

### 2.11. Study of ADMET and Drug Likeness

The ADMET and pharmacokinetic properties of the top ligands were predicted by inputting their canonical SMILES sequences into the admetSAR 2 (http://lmmd.ecust.edu.cn/admetsar2) [57] and SwissADME (http://www.swissadme.ch/index.php) servers [58]. The canonical SMILES for each lead molecule was obtained from the PubChem database. ADMET properties were determined for each ligand by utilizing various parameters, such as Lipinski's rule of five, BBB permeability, aqueous solubility, toxicity, and carcinogenicity. These characteristics significantly enhance the drug potential and effectiveness of phytochemicals in the treatment of diseases.

### 2.12. Molecular Dynamics (MD) Simulation

MD simulation is a widely used technique for exploring and validating the structural flexibility of protein–ligand complexes within a present time frame in a controlled environment. It can be applied effectively to reveal dynamic interactions and understand macromolecular structure-to-function relationships [59]. YASARA dynamics software was used to confirm the prediction results from the docking study [60]. Equilibration and production steps are included in the MD simulation, which starts after complete energy minimization [61]. To evaluate the structural integrity of the complexes, the GMFG protein was utilized as a control. The AMBER14 force field was applied in this simulation process, which is widely accepted [62]. The PME (particle-mesh Ewald) method was used to compute the long-range electrostatic interactions in the study, and the cut-off radius was fixed at 8 Å [63]. In this MD simulation, a 1.25 fs time step was applied, and the trajectories were saved after every 100 ps. The runtime of the MD simulation was 100 ns while keeping the pressure constant and employing the Berendsen thermostat [64]. By applying the TIP3P solvation model, a cubic simulation cell was generated, and periodic boundary conditions were maintained [65]. The overall environmental conditions for the system were established at a temperature of 298 K, pH 7.4, and a NaCl concentration of 0.9% [66]. The system was minimized by utilizing the steepest descent method [67]. The root-mean-square deviation (RMSD), root-mean-square fluctuation (RMSF), radius of gyration (RG), hydrogen bond number, and solvent-accessible surface area (SASA) were evaluated by analyzing the trajectory data from MD simulations [68].

## 3. Results

### 3.1. Data Processing and DEG Identification

**Figure 1:**
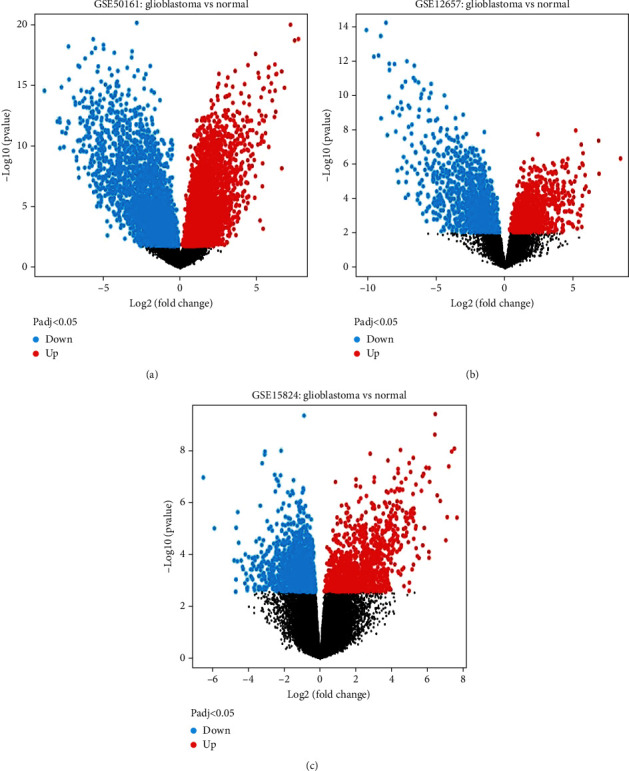
Volcano plot that depicts the upregulated and downregulated genes of the three datasets. (a) GSE50161, (b) GSE12657, and (c) GSE15824.

**Figure 2:**
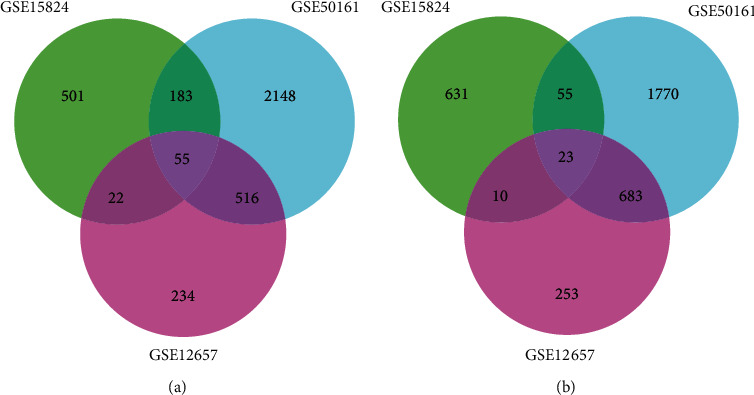
Common DEGs of the three datasets. (a) Upregulated DEGs. (b) Downregulated DEGs.

We analyzed the gene expression data of GBM to explore the DEGs. In comparison to those in normal patients, in the GSE50161 dataset, we found 5405 significant DEGs, 2902 of which were upregulated and 2503 of which were downregulated. This investigation also identified 1796 (827 upregulated and 969 downregulated) and 1479 (760 upregulated and 719 downregulated) DEGs in the GSE12657 and GSE15824 datasets, respectively. The upregulated genes and downregulated genes are visualized in Figures 1(a), 1(b), and 1(c). A comparative assessment of the three datasets revealed 55 common upregulated (Figure 2(a)) and 23 common downregulated DEGs (Figure 2(b)). These 78 common DEGs were subsequently subjected to PPI analysis.

### 3.2. Hierarchical Clustering Analysis

**Figure 3:**
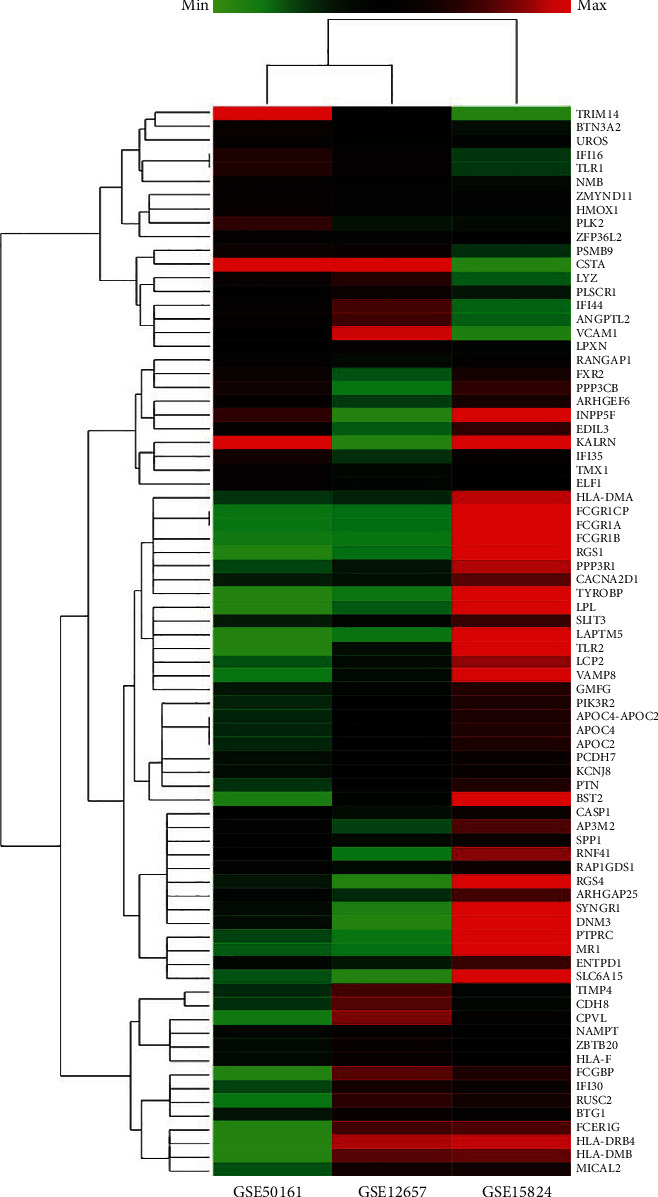
Hierarchical clustering analysis of the selected 78 genes common across targeted 3 datasets: GSE50161, GSE12657, and GSE15824.

We selected 78 genes for clustering analysis of gene expression data, chosen for their common presence across our targeted datasets.  Figure 3 presents a dendrogram depicting the hierarchical clustering of these selected genes. The color gradient illustrates the degree of gene expression regulation, with three primary color presets for effective visualization. Specifically, red indicates maximum or upregulated values, green represents minimum or downregulated values, and black signifies neutral or zero values, indicating no difference in expression. Dark green gradient colors denote negative values close to zero, while dark red gradient colors represent positive values near zero. As values approach zero, the gradient becomes darker.

### 3.3. PPIs and Identification of Hub DEGs

**Figure 4:**
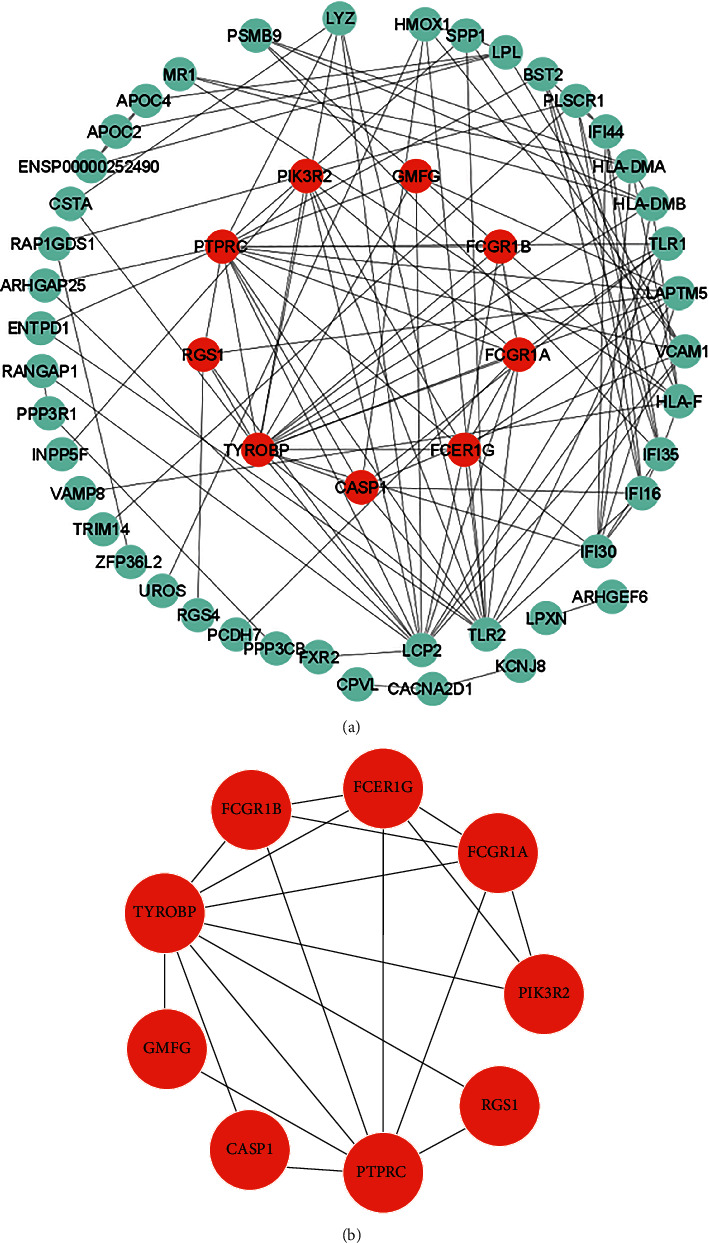
PPI network for identifying the hub DEGs. (a) Protein-protein interactions of the common DEGs. (b) Network of hub DEGs determined by the MCODE algorithm.

We explored PPI networks predicted by the common DEGs related to this disease to understand their functional interactions. The PPI pair contains 76 nodes and 29 edges, and the enrichment *p* value for the PPI is less than 1.0e − 16. Only interconnected nodes and edges are depicted in Figure 4(a). Moreover, several related nodes in the PPI networks were identified as hub genes. A topological analysis of the networks revealed 9 hub proteins for the common DEGs by using the MCODE algorithm, as shown in Figure 4(b). A list of the hub DEGs is presented in Table 2.

### 3.4. Analysis of Gene Ontologies (GO) and Pathway Enrichment

After determining the hub DEGs associated with the disease, we conducted significant GO and pathway enrichment analyses using curated databases to investigate the gene ontologies and pathways. Most of the DEGs were involved in the innate immune system. A wide range of GO terms and signaling pathways were enriched. The findings of the pathway analysis showed that the genes were mostly linked to several immune system processes. The top 10 biological processes, molecular functions, cellular components, and pathways are enumerated in Tables 3 and 4.

### 3.5. Regulatory Signatures Revealing Significant TFs

We identified 10 TFs associated with the targeted DEGs to uncover regulatory biomolecules that are likely to govern the expression of dysregulated genes at the posttranscriptional level (Table 5). Significant TFs, including Pax6, PPARG, INSM1, RREB1, CTCF, REST, NHLH1, RXR::RAR, DR5, RXRA::VDR, and PLAG1, were found to play significant roles in the regulation of the DEGs identified in this study.

### 3.6. Survival Analysis

**Figure 5:**
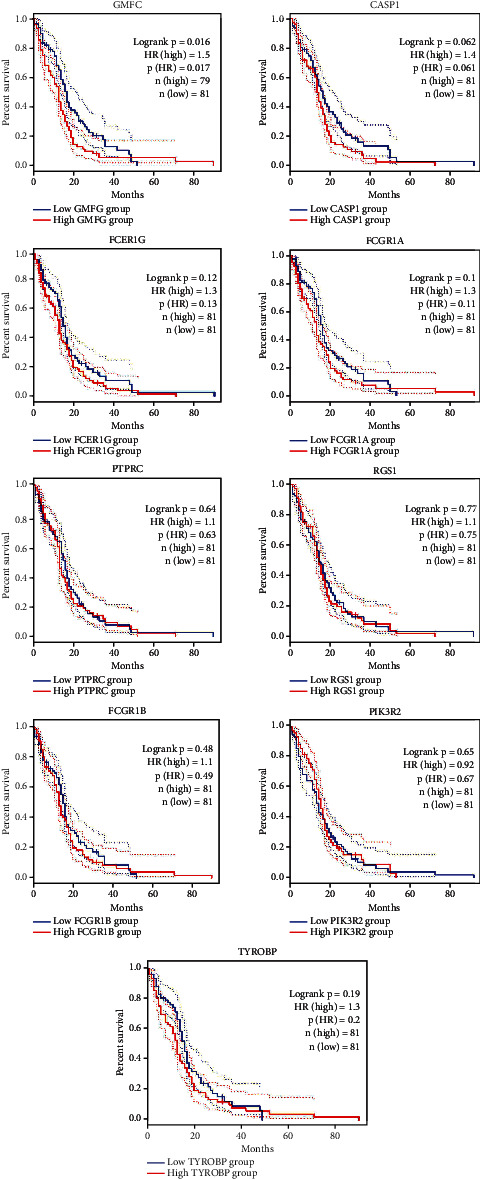
Survival analysis of the hub DEGs in glioblastoma. *GMFG* was significant (*p* value < 0.05). The remaining plots were nonsignificant (*p* value > 0.05).

The correlation between the level of *GMFG* expression and overall survival (OS) in GBM patients was analyzed via the Kaplan–Meier plots. According to the *p* value, there were no correlations between the gene expression of eight genes and OS, with the exception of *GMFG.* These findings suggest that high expression of *GMFG* is significantly associated with poor OS (Figure 5).

### 3.7. Validation of the *GMFG* Gene

**Figure 6:**
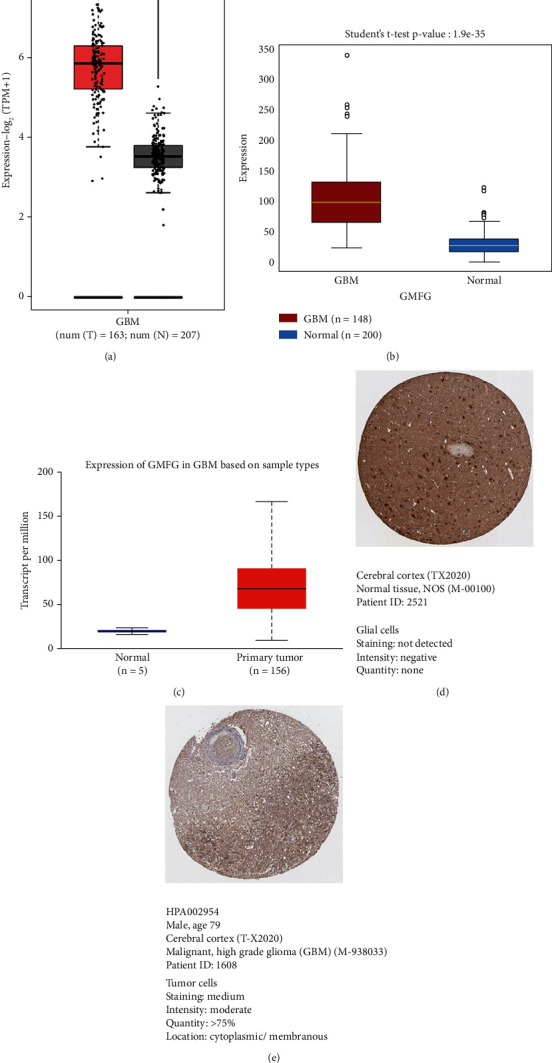
The mRNA expression level of the *GMFG* gene in GBM tissues. (a) GEPIA2 server. The black and red boxes represent normal and cancerous samples, respectively (log2 transformation was used to normalize the results). (b) OncoDB server. (c) UALCAN server. Based on immunohistochemistry data, the protein expression levels of *GMFG* in (d) normal tissue (glial cells) and (e) GBM (tumor cells) are provided.

The *GMFG* gene was studied to determine the extent to which it was expressed at the mRNA level. Analysis via the GEPIA 2.0 server revealed higher expression of *GMFG* mRNA in GBM tissues (normal: 207; tumor: 163) than in normal tissues (Figure 6(a)). The *GMFG* gene was subsequently evaluated on the OncoDB web server to determine its expression patterns in malignant tissues. Like in previous results, *GMFG* mRNA was more highly expressed in GBM tissues (*p* = 1.9e − 35) than in normal samples (Figure 6(b)). Afterwards, cross-check analysis via the UALCAN server similarly revealed greater expression of GMFG mRNA in GBM tissues than in normal tissues (Figure 6(C)). Next, we examined the expression of GMFG protein in normal glial cells and GBM tumor cells using immunohistochemistry data archived from the Human Protein Atlas Project. We detected no staining in normal glial cells, whereas tumor cells exhibited a moderate staining level. Additionally, the intensity signal in glial cells was negative, while tumor cells showed a moderate intensity (quantity > 75%) signal (Figures 6(d) and 6(e)).

### 3.8. Molecular Docking Analysis

**Figure 7:**
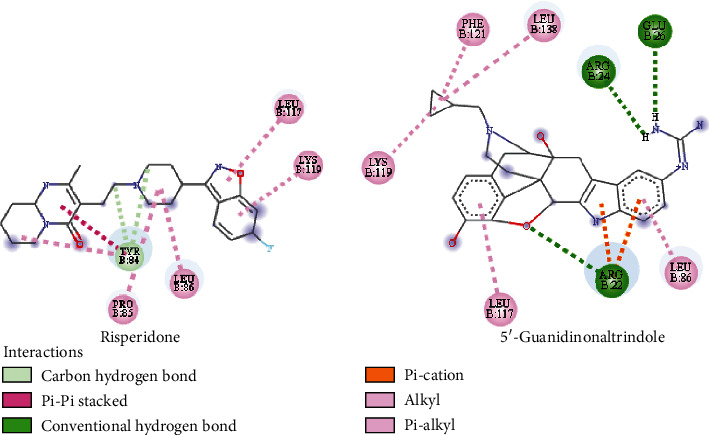
Two-dimensional (2D) binding modes and docking interactions of selected ligands with the GMFG protein (PDB ID: 3I50).

**Figure 8:**
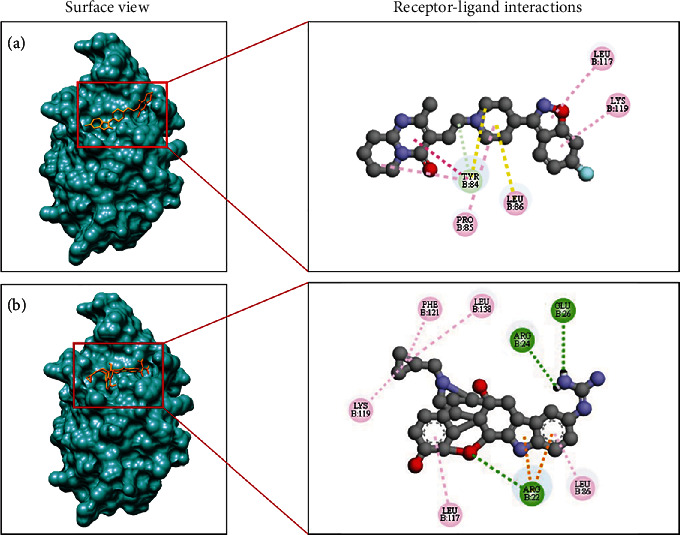
Surface view of the docked complexes for the top two compounds and their receptor–ligand interactions. (a) Risperidone and (b) 5′-guanidinonaltrindole.

The docking of the GMFG target with each of the selected compounds was accomplished successfully. A lower binding energy indicates stronger binding affinity; consequently, the molecules exhibiting the lowest binding energy were identified as the most potent ligands for inhibiting the target receptor. Through an examination of the docking interactions, we observed that amcinonide, irinotecan, folic acid, methotrexate, risperidone, pancuronium, epigallocatechin gallate, daunorubicin, zafirlukast, and 5′-guanidinonaltrindole had the lowest binding scores, at −8.9, −8.9, −8.6, −8.6, −8.5, −8.5, −8.5, −8.5, −8.5, and −8.4 kcal/mol, respectively. Following the completion of the molecular docking analysis, this study assessed the ADMET properties and drug-likeness characteristics. Consequently, two compounds, namely, risperidone and 5′-guanidinonaltrindole, were identified as potential inhibitors of the GMFG protein. The GMFG-risperidone interaction formed a carbon hydrogen and Pi-Pi stacked bond with TYR84; an alkyl bond with PRO85 and LEU86; and a pi-alkyl bond with TYR84, LEU117, and LYS119. Conversely, the GMFG-5′-guanidinonaltrindole interaction formed conventional hydrogen bonds with ARG22, ARG24, and GLU26; pi-cation interactions with ARG22; pi-alkyl interactions with LEU86, LEU117, and PHE121; and alkyl interactions with LYS119 and LEU138 (Figure 7). A surface view and receptor–ligand interactions of the docked complexes for the top two compounds within the active and catalytic sites of the GMFG protein are shown in Figure 8. The binding scores and types of interactions with the target protein can be found in Table 6.

### 3.9. ADMET Analysis

The various pharmacokinetic parameters of the top 10 identified compounds were evaluated based on Lipinski's Rule of Five. Risperidone and 5′-guanidinonaltrindole were selected because they showed no violation of Lipinski's Rule of Five. The ADMET properties of the selected compounds indicated ideal drug-like characteristics (Table 7). Considering factors such as the lowest binding affinity and pharmacokinetic properties, we selected the top two ligands, risperidone and 5′-guanidinonaltrindole, for further study.

### 3.10. MD Simulation

**Figure 9:**
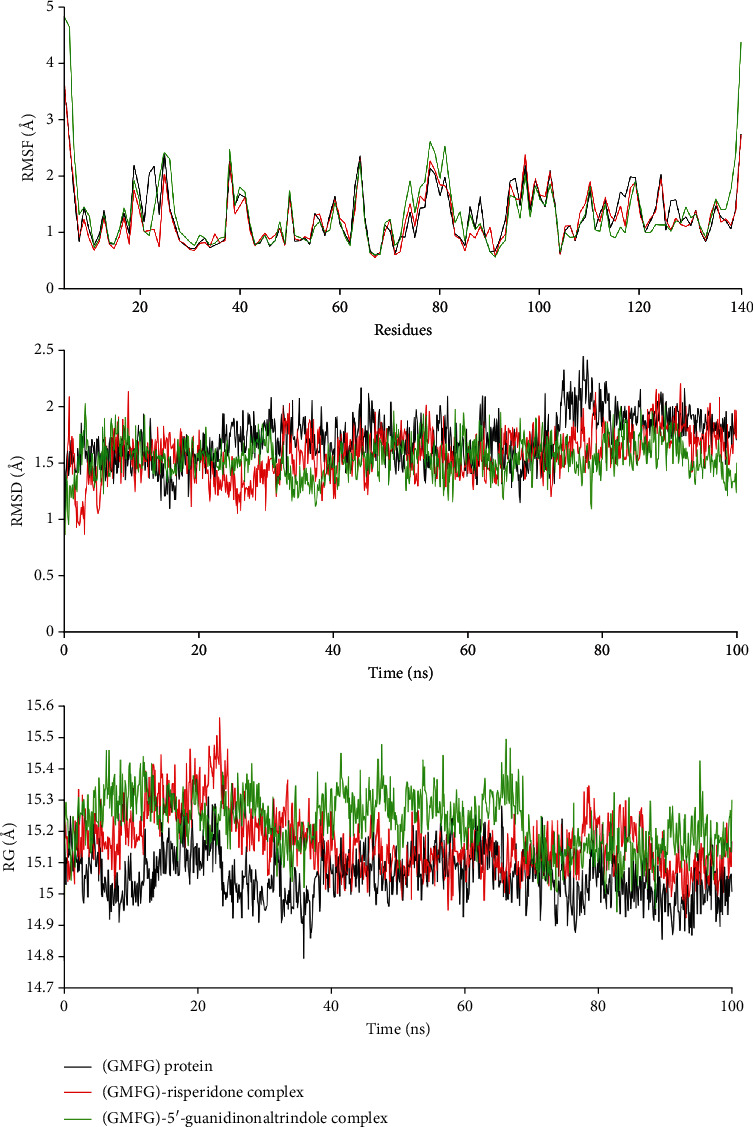
RMSF, RMSD, and RG analysis of the unligated GMFG protein and (GMFG)–risperidone/5′-guanidinonaltrindole complex. For each system, the MD simulations were performed for 100 ns.

**Figure 10:**
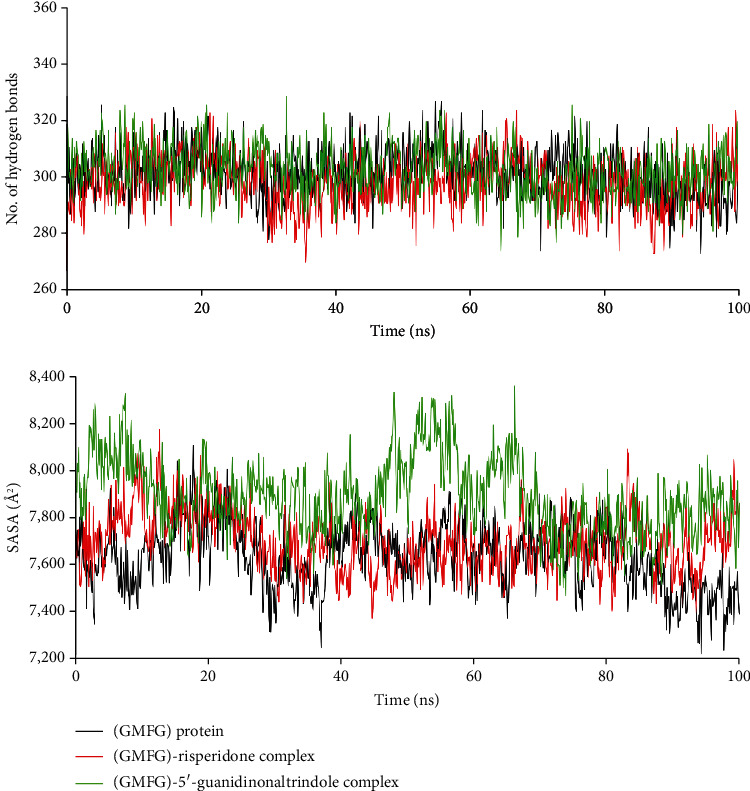
Analysis of the hydrogen bond number and SASA of unligated (GMFG) protein and (GMFG)–risperidone/5′-guanidinonaltrindole complex. For each system, MD simulations were performed for 100 ns.

MD simulation is an essential analysis of the structural stability and flexibility of biological macromolecules [69]. MD simulations were performed for 100 ns to investigate the conformational changes and binding mechanism of the (GMFG) protein, (GMFG)-risperidone complex, and (GMFG)-5′-guanidinonaltrindole complex. After 100 ns of simulation, dynamic trajectories were inspected, and parameters such as the RMSF, RMSD, and RG (Figure 9), as well as the number of hydrogen bonds and solvent accessible surface area (SASA) (Figure 10), were computed.

By analyzing the RMSF of the (GMFG) protein, (GMFG)-risperidone complex, and (GMFG)-5′-guanidinonaltrindole complex, it was observed that the protein and the two complexes displayed an overall similar range of fluctuations. These findings suggested that the two complexes exhibited desirable protein residue flexibility; here, the fluctuation range of the protein was considered a control. To check the firmness of the GMFG protein, the GMFG-risper complex, and the GMFG-5′-guanidinonaltrindole complex, the RMSD values of the C*α* atoms of these compounds were assessed [70]. The GMFG protein slightly fluctuated from 75 to 81 ns. However, all the other trajectories of the protein remained stable. However, from the start to the end, all the trajectories of both the (GMFG)-risperidone complex and the (GMFG)-5′-guanidinonaltrindole complex exhibited an equilibrium state, which suggested the strong stability of both protein–ligand complexes with respect to the reference protein (GMFG).

The structural compactness and Rg were evaluated [71]. The GMFG- risperidone complex was stable at the starting position. However, from 8 to 38 ns, several small fluctuations were observed. After that, the equilibrium state was reached. The (GMFG)-5′-guanidinonaltrindole complex also showed initial stability. However, from 7 to 70 ns, several fluctuations occurred. Afterwards, the material also exhibited stability. Based on the (GMFG) protein Rg trajectory pattern as a control, it was observed that the (GMFG)-risperidone complex had a better Rg profile than the (GMFG)-5′-guanidinonaltrindole complex.

The GMFG protein, (GMFG)-risperidone complex, and (GMFG)-5′-guanidinonaltrindole complex had similar numbers of hydrogen bonds, which suggests the desirable integrity of these compounds. SASA was analyzed to evaluate folding and stability [72]. The (GMFG)-risperidone complex exhibited a fluctuating nature from 5 to 11 ns. Afterwards, the system reached a more or less stable equilibrium state, which indicated the considerable compactness of the compound. In contrast, the (GMFG)-5′-guanidinonaltrindole complex exhibited high fluctuations from 2 to 10 ns and 46 to 58 ns, which indicated that it had slightly decreased stability. Based on the GMFG protein SASA trajectories as a reference, the (GMFG)-risperidone complex was shown to have a better SASA profile or compactness than the (GMFG)-5′-guanidinonaltrindole complex.

**Figure 11:**
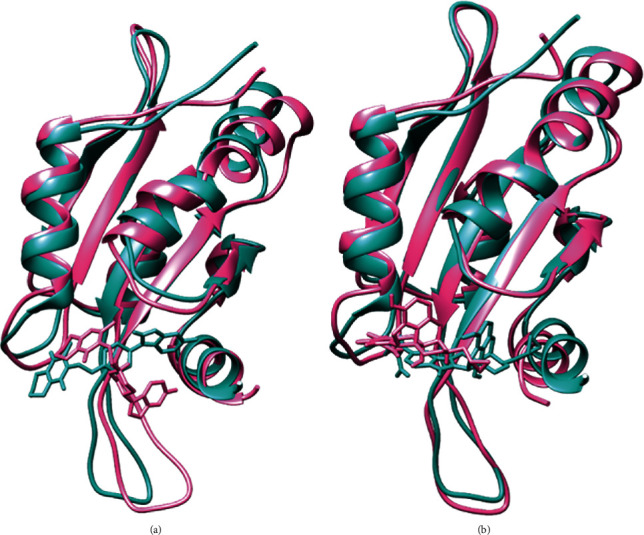
The superimposed view of pre and postmolecular dynamics simulation structures of (a) risperidone and (b) 5′-guanidinonaltrindole. The light in green denotes the premolecular dynamic structure, and the hot pink denotes the postmolecular dynamic structure.

**Figure 12:**
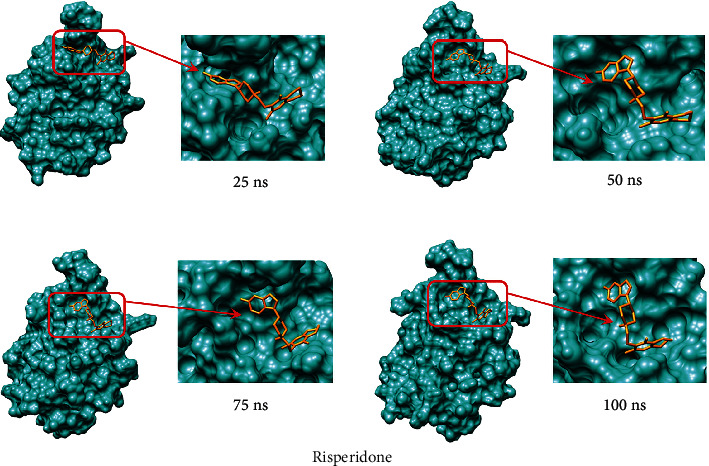
Surface view of the risperidone and GMFG protein complex after 25, 50, 75, and 100 ns of MD simulation. The figure shows the structural change every 25 ns.

**Figure 13:**
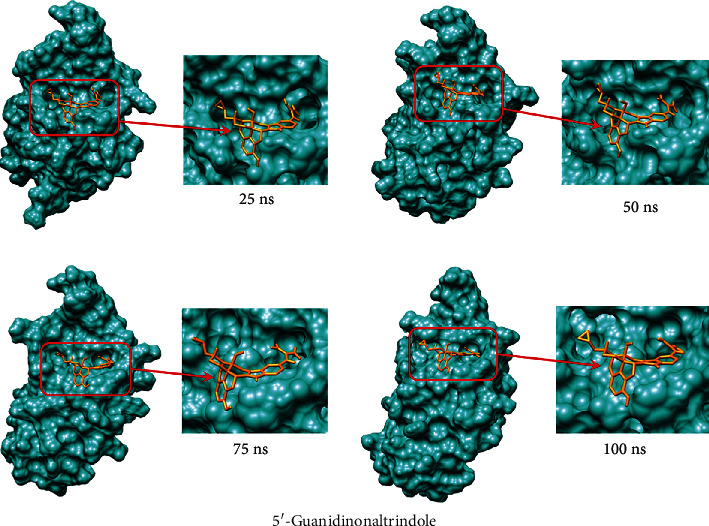
Surface view of the 5′-guanidinonaltrindole and GMFG protein complex after 25, 50, 75, and 100 ns of MD simulation. The figure shows the structural change every 25 ns.

Analysis of these parameters indicated that despite the two complexes showing potentiality and very close outcomes, the (GMFG)-risperidone complex is considered a more promising candidate due to its better Rg and SASA profile. As determined by MD simulation, minor conformational changes occurred in these two complexes, similar to what was observed for the GMFG reference protein. To demonstrate the changes in the binding cavity, the superimposition of pre- and post-MD simulation structures of risperidone and 5′-guanidinonaltrindole was assessed (Figure 11). The structural changes in risperidone and 5′-guanidinonaltrindole were observed after every 25 ns. A snapshot was taken of the surface view of the (GMFG)-risperidone complex and the (GMFG)-5′-guanidinonaltrindole complex at 25, 50, 75, and 100 ns (Figures 12 and 13).

## 4. Discussion

GBM is an aggressive and highly malignant brain tumor with limited treatment options and a poor prognosis. Over the years, significant efforts have been made to better understand the molecular mechanisms underlying GBM development and progression, leading to the exploration of novel treatment strategies. One area of research involves the exploration of molecular and genetic alterations in GBM, intending to identify specific biomarkers and therapeutic vulnerabilities. For instance, studies have revealed the role of genetic mutations in genes such as IDH1, EGFR, TP53, and PTEN in GBM development and progression [1]. Additionally, advancements in genomic sequencing techniques have enabled comprehensive profiling of GBM tumors, aiding in the identification of potentially targetable mutations [15]. Furthermore, immunotherapy approaches, including immune checkpoint inhibitors and personalized vaccines, are being investigated as promising strategies to harness the immune system's response against GBM [73]. Despite numerous research efforts, the precise mechanisms driving the development of GBM have not been fully elucidated. Consequently, there is a pressing need for further research to uncover potential biomarkers and develop more efficient treatment options for GBM, aiming to improve the prognosis and OS rates of individuals affected by this highly destructive condition.

In the present study, we analyzed the gene expression data of GBM patients to identify DEGs, hub DEGs, genes related to biological activities, molecular pathways, regulatory biomolecules, and potential biomarkers. This was performed utilizing a multiomics data integration framework to identify potential therapeutic targets for GBM. By analyzing the patterns of gene expression, we found 8680 DEGs, 4697 of which were upregulated and 3983 of which were downregulated. Comparative analysis of the datasets identified 55 upregulated and 23 downregulated DEGs. Moreover, PPI analysis revealed nine hub DEGs, which encode significant hub proteins that exhibit strong interconnections. These hub DEGs were found to be closely associated with various biological activities and pathways in the functional enrichment study. These activities and pathways included the Fc receptor-mediated stimulatory signaling pathway, the Fc-gamma receptor signaling pathway, myeloid cell activation involved in the immune response, neutrophil activation, neutrophil degranulation, and neutrophil activation involved in the immune response, regulation of B-cell proliferation, and apoptosis.

The identification of hub DEGs and the regulatory network commonly resulted in the identification of 10 major transcriptional regulators (Pax6, PPARG, INSM1, RREB1, CTCF, REST, NHLH1, RXR::RAR, DR5, RXRA::VDR, and PLAG1) for the hub DEGs. These TFs are believed to play a significant role in the regulation of gene expression in GBM and are considered to be major regulators of this disease. Survival analysis indicated that high expression of the *GMFG* gene was significantly associated with shorter OS than was high expression of other hub DEGs. Therefore, overexpression of the *GMFG* gene may lead to unfavorable outcomes in GBM patients. Consequently, we validated these DEGs as potential prognostic biomarkers.

Initially, at the mRNA level, the GMFG gene was shown to be differentially expressed (upregulated) in GBM tissues, indicating its potential tumourigenic role in the formation and development of GBM. Further, we conducted a comparative study on immunohistochemistry between GBM and normal cells. In this investigation, we observed significant differences in staining, intensity, and quantity between tumor cells and normal glial cells. In a previous study, it was observed that *GMFG* exhibited higher expression levels in GBM tissues [74]. This consistency across studies enhances the credibility of the results and strengthens the argument for the role of GMFG in GBM. Since cancer formation is a complex process that varies depending on the subtype, grade, and demographics of the cancer [75], it is necessary to analyze the expression level of a gene across different variables to understand its function in disease development. Then, we evaluated the efficacy of the GMFG protein as a drug target using molecular docking and dynamics studies.

Molecular docking is a valuable technique that involves the binding of a small molecule (ligand) to the binding site of a target receptor to demonstrate its attachment. In this study, a total of 692 ligand molecules were selected to predict inhibitors of the GMFG protein that are associated with the development of GBM. The ligands were docked against the target receptor to evaluate the anti-GMFG potential of the ligands, and therefore, the two ligands with the best docking were chosen for further analyses. The inhibitory efficacy of the ligands on the receptor was determined by the lower binding affinity score. This study demonstrated that risperidone and 5′-guanidinonaltrindole have strong affinities for GMFG, as indicated by binding scores of −8.5 and −8.4 kcal/mol, respectively. Risperidone is an antipsychotic medicine that is commonly used to treat a variety of psychiatric conditions, including schizophrenia [76], bipolar disorder [77], Alzheimer's disease [78], and dementia [79]. Though the risperidone is an antipsychotic drug, it has been shown to have a therapeutic effect on GBM [80]. This compound was also reported to have a potential effect for lung cancer [81] and gastric cancer [82]. This drug molecule forms multiple nonbonded interactions with the GMFG protein and interacts with the TYR84, PRO85, LEU86, LEU117, and LYS119 residues. 5′-Guanidinonaltrindole is an opioid antagonist that has been shown in animal experiments to have antidepressant effects [83, 84]. Specifically, it is a selective kappa-opioid receptor (KOR) antagonist that has shown promise in cancer treatment through its ability to modulate various signaling pathways associated with tumor growth and metastasis [85]. 5′-Guanidinonaltrindole interacted with the ARG22, ARG24, GLU26, LEU86, LEU117, LYS119, PHE121, and LEU138 residues of the GMFG protein. The results of the Lipinski filter, SwissADME, and admetsar2 analyses demonstrated that the selected top ligand molecules also overcame the drug-like requirements. These evaluations highlighted the possible pharmacokinetic properties of these compounds.

Estimation of pharmacokinetic properties facilitates drug development. The permeability of a drug through biological barriers is influenced by factors such as the topological polar surface area (TPSA) and molecular weight. A larger molecular weight and TPSA are linked to lower drug permeability. BBB penetration is necessary for the use of medications to treat the brain. The selected ligand molecules have the ideal molecular weight, TPSA, optimum number of H-bond donors and acceptors, and ability to penetrate the BBB. Additionally, the toxicity profiles of the two compounds showed that the drug molecules have no carcinogenicity or toxicity, suggesting their potency as drugs.

MD simulation is a powerful tool for assessing postmolecular docking analysis and validation [86]. Risperidone and 5′-guanidinonaltrindole were the selected compounds after docking, and ADMET analysis was performed. Therefore, the (GMFG)-risperidactam (risperidone) complex and the (GMFG)-5′-guanidinonaltrindole complex were evaluated via 100 ns MD simulation, where the simulation of the GMFG protein was used as a reference. The parameters of the RMSF, RMSD, RG, hydrogen bond number, and SASA were analyzed thoroughly. Both complexes were stable following the reference protein. However, the (GMFG)-risperidone complex displayed more desirable Rg and SASA results than did the (GMFG)-5′-guanidinonaltrindole complex. Therefore, risperidone is most likely the best inhibitor of the target protein.

The tendency of the *GMFG* gene to be overexpressed and its effect on survival patterns suggest that the *GMFG* gene can be used to diagnose and treat GBM. Overall, the findings of this empirical study acquired from differential expression analysis, promoter methylation, and mutation rate of the *GMFG* gene in GBM tissues indicate that this gene is most likely to play a crucial role in the development and treatment of GBM. Finally, this study suggested that the *GMFG* is likely to serve as a viable prognostic and therapeutic target for GBM. The clinical relevance of GMFG as a prognostic biomarker and therapeutic target needs to be validated in larger and independent cohorts of GBM patients. The actual biological activity and therapeutic efficacy of the identified compounds need to be confirmed through laboratory and clinical experiments. Although our molecular docking and dynamics simulation substantiated a preliminary result of the interaction between GMFG and the candidate compounds, these finding needs to be validated by clinical trials with GBM patients.

## 5. Conclusion

Analysis of the hub DEGs by GO and pathway enrichment revealed the important processes and pathways involved in GBM. Furthermore, this study demonstrated a significant correlation between the overexpression of the *GMFG* gene and OS in GBM patients. Our research also revealed that the expression level of *GMFG* is high in GBM tissues, suggesting that *GMFG* is a potential diagnostic and therapeutic target for GBM. Risperidone and 5′-guanidinonaltrindole exhibited significant docking energy when interacting with two lead molecules. The MD simulation also validated the stability of the interaction between the active pocket of the protein and the compounds. Overall, our findings revealed that risperidone is a potential target for GBM treatment. This investigation holds promise for advancing the understanding and management of GBM and, thus, can aid in disease treatment and drug development.

## Figures and Tables

**Table 1:** Details of the selected datasets, including GEO accession numbers, platforms, case and control samples, and DEG counts.

**GEO accessions**	**Platforms**	**Case samples**	**Control samples**	**Upregulated DEGs**	**Downregulated DEGs**	**Total DEGs**
GSE50161	GPL570	34	13	2902	2530	5432
GSE12657	GPL8300	7	5	827	969	1796
GSE15824	GPL570	15	5	760	719	1479

**Table 2:** The predicted hub DEGs with their designation.

**Genes**	**Elaboration**
FCER1G	Fc epsilon receptor Ig
FCGR1A	Fc gamma receptor Ia
FCGR1B	Fc gamma receptor Ib
PIK3R2	Phosphoinositide-3-kinase regulatory subunit 2
RGS1	Regulator of G-protein signaling 1
PTPRC	Protein tyrosine phosphatase receptor type C
CASP1	Caspase-1
GMFG	Glia maturation factor gamma
TYROBP	TYRO protein tyrosine kinase-binding protein

**Table 3:** Gene Ontology analysis of the hub DEGs of glioblastoma. The top 10 terms of each category are listed herewith.

**Categories**	**GO ID**	**Terms**	**p** **value**	**Genes**
Biological process	GO:0038094	Fc-gamma receptor signaling pathway	0.000004	FCER1G; PIK3R2; FCGR1A
	GO:0002431	Fc receptor-mediated stimulatory signaling pathway	0.000004	FCER1G; PIK3R2; FCGR1A
	GO:0042119	Neutrophil activation	0.000022	FCER1G; TYROBP
	GO:0002275	Myeloid cell activation involved in immune response	0.000031	FCER1G; TYROBP
	GO:0043312	Neutrophil degranulation	0.000038	FCER1G; PTPRC; TYROBP; GMFG
	GO:0002283	Neutrophil activation involved in immune response	0.000039	FCER1G; PTPRC; TYROBP; GMFG
	GO:0002446	Neutrophil-mediated immunity	0.000040	FCER1G; PTPRC; TYROBP; GMFG
	GO:0061097	Regulation of protein tyrosine kinase activity	0.000132	PTPRC; FCGR1A
	GO:0030217	T-cell differentiation	0.000146	FCER1G; PTPRC
	GO:0030888	Regulation of B-cell proliferation	0.000184	PTPRC; TYROBP

Molecular function	GO:0019864	IgG binding	0.0022	FCER1G
	GO:0097199	Cysteine-type endopeptidase activity involved in apoptotic signaling pathway	0.0045	CASP1
	GO:0097200	Cysteine-type endopeptidase activity involved in execution phase of apoptosis	0.0058	CASP1
	GO:0008656	Cysteine-type endopeptidase activator activity involved in apoptotic process	0.0067	CASP1
	GO:0097153	Cysteine-type endopeptidase activity involved in apoptotic process	0.0067	CASP1
	GO:0046935	1-Phosphatidylinositol-3-kinase regulator activity	0.0072	PIK3R2
	GO:0019198	Transmembrane receptor protein phosphatase activity	0.0072	PTPRC
	GO:0005001	Transmembrane receptor protein tyrosine phosphatase activity	0.0072	PTPRC
	GO:0050700	CARD domain binding	0.0072	CASP1
	GO:0016505	Peptidase activator activity involved in apoptotic process	0.0076	CASP1

Cellular component	GO:0030667	Secretory granule membrane	0.0002	FCER1G; PTPRC; TYROBP
	GO:0032059	Bleb	0.0022	PTPRC
	GO:0005887	Integral component of plasma membrane	0.0026	FCER1G; PTPRC; TYROBP; FCGR1A
	GO:0101002	Ficolin-1-rich granule	0.0029	FCER1G; GMFG
	GO:0030659	cytoplasmic vesicle membrane	0.0119	PTPRC; TYROBP
	GO:0009898	Cytoplasmic side of plasma membrane	0.0245	PTPRC
	GO:0101003	Ficolin-1-rich granule membrane	0.0271	FCER1G
	GO:0030669	Clathrin-coated endocytic vesicle membrane	0.0306	FCGR1A
	GO:0070821	Tertiary granule membrane	0.0324	FCER1G
	GO:0045334	Clathrin-coated endocytic vesicle	0.0376	FCGR1A

**Table 4:** Pathway analysis of the common DEGs. The top 10 terms of each category are listed.

**Categories**	**Terms**	**p** **value**	**Genes**
Reactome	Innate immune system R-HSA-168249	0.00000003	FCER1G; PTPRC; TYROBP; GMFG; CASP1; PIK3R2; FCGR1A
	Immune system R-HSA-168256	0.00000244	FCER1G; PTPRC; TYROBP; GMFG; CASP1; PIK3R2; FCGR1A
	Other semaphorin interactions R-HSA-416700	0.00003066	PTPRC; TYROBP
	Neutrophil degranulation R-HSA-6798695	0.00003397	FCER1G; PTPRC; TYROBP; GMFG
	Role of phospholipids in phagocytosis R-HSA-2029485	0.00005817	PIK3R2; FCGR1A
	DAP12 signaling R-HSA-2424491	0.00006763	TYROBP; PIK3R2
	GPVI-mediated activation cascade R-HSA-114604	0.00008866	FCER1G; PIK3R2
	DAP12 interactions R-HSA-2172127	0.00016099	TYROBP; PIK3R2
	Adaptive immune system R-HSA-1280218	0.00019458	PTPRC; TYROBP; PIK3R2; FCGR1A
	Semaphorin interactions R-HSA-373755	0.00035767	PTPRC; TYROBP

**Table 5:** Top regulatory molecules (TFs) for common DEGs of glioblastoma.

**TFs**	**p** **value**	**Genes**
Pax6	0.0025	PIK3R2; FCGR1B
PPARG	0.0122	FCER1G; TYROBP; GMFG; PIK3R2; FCGR1B
INSM1	0.0156	FCGR1B
RREB1	0.0156	FCGR1B
CTCF	0.0156	FCGR1B
REST	0.0156	FCGR1B
NHLH1	0.0156	FCGR1B
RXR::RAR	0.0156	FCGR1B
RXRA::VDR	0.0156	FCGR1B
PLAG1	0.0156	FCGR1B

**Table 6:** Docking interactions of risperidone and 5′-guanidinonaltrindole with the GMFG protein.

**Name of the ligand**	**Binding affinity (kcal/mol)**	**Interacting amino acids**	**Types**	**Bond distance in Å**	**Type of interaction**
Risperidone	−8.5	TYR84	Hydrogen bond	3.70	Carbon hydrogen bond
			Hydrogen bond	3.64	Carbon hydrogen bond
			Hydrophobic	5.67	Pi-Pi stacked
			Hydrophobic	4.90	Pi-alkyl
		PRO85	Hydrophobic	5.41	Alkyl
		LEU86	Hydrophobic	5.21	Alkyl
		LEU117	Hydrophobic	5.02	Pi-alkyl
		LYS119	Hydrophobic	5.20	Pi-alkyl

5′-Guanidinonaltrindole	−8.4	ARG22	Hydrogen bond	2.96	Conventional hydrogen bond
		ARG22	Electrostatic	3.68	Pi-Cation
		ARG22	Hydrogen bond; Electrostatic	3.31	Pi-cation; Pi-donor hydrogen bond
		ARG24	Hydrogen bond	2.74	Conventional hydrogen bond
		GLU26	Hydrogen bond	3.06	Conventional hydrogen bond
		LEU86	Hydrophobic	5.42	Pi-alkyl
		LEU117	Hydrophobic	5.28	Pi-alkyl
		LYS119	Hydrophobic	4.16	Alkyl
		PHE121	Hydrophobic	5.10	Pi-alkyl
		LEU138	Hydrophobic	5.29	Alkyl

**Table 7:** Pharmacological profile and drug-likeness studies of the top 10 potential compounds for GMFG targets.

**Compounds**	**MW (g/mol)**	**Num. H-bond acceptors**	**Num. H-bond donors**	**MlogP**	**Molar refractivity**	**TPSA (Å** ^**2**^ **)**	**Blood–brain barrier**	**Aqueous solubility (LogS)**	**Toxicity**	**Carcinogens**	**Violation**
Amcinonide	502.57	8	1	2.37	127.74	99.13	BBB+	−4.9847	None	None	1
Irinotecan	586.68	8	1	2.55	169.63	114.2	BBB+	−3.4931	None	None	1
Folic acid	441.4	9	6	-0.62	111.92	213.28	BBB+	−3.3434	None	None	2
Risperidone	410.48	6	0	3.19	117.71	64.16	BBB+	−3.3048	None	None	0
Methotrexate	454.44	9	5	-1.13	118.4	210.54	BBB-	−3.0651	None	None	1
Pancuronium	572.86	6	4	-2.3	175.46	52.6	BBB+	−3.0774	None	None	1
Epigallocatechin gallate	458.37	11	8	-0.44	112.06	197.37	BBB-	−3.3141	None	None	2
5′-Guanidinonaltrindole	471.55	5	5	2.04	136.6	133.12	BBB+	−3.0546	None	None	0
Daunorubicin	527.52	11	5	-1.35	131.5	185.84	BBB-	−3.378	Toxic	None	2
Zafirlukast	575.68	6	2	3.1	157.82	124.11	BBB+	−3.8469	None	None	1

## Data Availability

All data generated or analyzed during this study are included in this manuscript.

## Nomenclature

ANOVA: analysis of variance
BBB: blood–brain barrier
BP: biological process
CC: cellular component
CNAs: copy number alterations
CNS: central nervous system
DEGs: differentially expressed genes
EGFR: epidermal growth factor receptor
fs: femtosecond
GBM: glioblastoma
GBM: glioblastoma multiforme
GEO: Gene Expression Omnibus
GMFG: glia maturation factor gamma
GO: Gene Ontology
GTEx: genotype-tissue expression
IDH: isocitrate dehydrogenase
MCODE: molecular complex detection
MD: molecular dynamics
MF: molecular function
NF1: neurofibromatosis type 1
ns: nanosecond
PDGFRA: platelet-derived growth factor receptor alpha
PPI: protein-protein interactions
ps: picosecond
PTEN: phosphatase and TENsin homolog deleted on chromosome 10
RG: radius of gyration
RMSD: root mean square deviation
RMSF: root-mean-square fluctuation
SASA: solvent accessible surface area
TCGA: The Cancer Genome Atlas
TF: transcription factors
TPSA: topological polar surface area
VEGF: vascular endothelial growth factor
